# Discovery of novel secretome CAZymes from *Penicillium sclerotigenum* by bioinformatics and explorative proteomics analyses during sweet potato pectin digestion

**DOI:** 10.3389/fbioe.2022.950259

**Published:** 2022-09-16

**Authors:** Kristian Barrett, Hai Zhao, Pengfei Hao, Antony Bacic, Lene Lange, Jesper Holck, Anne S. Meyer

**Affiliations:** ^1^ Department of Biotechnology and Biomedicine, Technical University of Denmark, Kgs. Lyngby, Denmark; ^2^ Chengdu Institute of Biology, Chinese Academy of Science, Chengdu, China; ^3^ La Trobe Institute for Agriculture and Food, La Trobe University, Melbourne, VIC, Australia; ^4^ LLa BioEconomy, Research & Advisory, Valby, Denmark

**Keywords:** sweet potato tuber, enzymatic extraction, pectin, fungal proteome, enzyme discovery

## Abstract

Novel selective enzymatic refining of sweet potato processing residues requires judicious enzyme selection and enzyme discovery. We prepared a pectinaceous cell wall polysaccharide fraction from sweet potato using an enzymatic a treatment to preserve the natural linkages and substitutions. Polysaccharide composition and linkage analysis data confirmed the pectinaceous polysaccharide fraction to be a rhamnogalacturonan I-rich fraction with a high content of arabinogalactan Type I. We hypothesized that the post-harvest tuber pathogenic fungus *Penicillium sclerotigenum* would harbor novel enzymes targeting selective sweet potato pectin modification. As part of the study, we also report the first genome sequence of *P. sclerotigenum*. We incubated the sweet potato pectinaceous fraction with *P. sclerotigenum*. Using proteomics accompanied by CUPP-bioinformatics analysis, we observed induced expression of 23 pectin-associated degradative enzymes. We also identified six abundantly secreted, induced proteins that do not correspond to known CAZymes, but which we suggest as novel enzymes involved in pectin degradation. For validation, the predicted CUPP grouping of putative CAZymes and the exo-proteome data obtained for *P. sclerotigenum* during growth on sweet potato pectin were compared with proteomics and transcriptomics data reported previously for pectin-associated CAZymes from *Aspergillus niger* strain NRRL3. The data infer that *P. sclerotigenum* has the capacity to express several novel enzymes that may provide novel opportunities for sweet potato pectin modification and valorization of sweet potato starch processing residues. In addition, the methodological approach employed represents an integrative systematic strategy for enzyme discovery.

## Introduction

Sweet potato (*Ipomoea batatas* L.) is the second most abundant tuberous crop in the world, after cassava, with an annual production of 89.5 million tons, with almost two-thirds, i.e., 49 million tons, coming from China alone (FAOSTAT, 2022). The consumption of sweet potato as a staple food is declining, and the primary usage of sweet potato tubers is now for animal feed and for industrial starch production. In China, new types of sweet potato products have been commercialized as healthy snacks and food supplements, and 10% of the annual sweet potato production is now processed for starch manufacturing (Lareo et al., 2013; El Sheikha and Ray, 2017). During this processing, most of the starch is extracted by mechanical rasping and washing treatment. For each ton of starch produced, 4.5–5.0 tons of wet processing pulp is generated (Arachchige et al., 2021). This processing pulp is left unexploited, and since the pulp has a high biochemical oxygen demand (BOD), the large quantities are considered an environmental burden (Arachchige et al., 2021).

The industrial processing pulp resulting from the production of sweet potato starch mainly comprises the tuber cell wall material, which is rich in pectin and bioactive compounds that have presumed health beneficial effects i.e., prebiotic, anti-cancer, and anti-diabetic potential (Holck et al., 2014; Wang et al., 2016). In the research literature, the pulp has been shown to possess beneficial effects after lactic acid bacterial fermentation (Zhu et al., 2022), but the potentially beneficial compounds, notably the pectinous fibers, have mostly been chemically extracted to study and potentially exploit their bioactive effects (Leclere et al., 2013; Zhang et al., 2013; Ogutu et al., 2018; Liu et al., 2021). However, enzymatic extraction offers a more gentle and selective way of obtaining structurally intact carbohydrate structures from the pulp than chemical extraction. Indeed, enzymatic release of specific, high molecular weight bifidogenic and highly fermentable galactan-rich rhamnogalacturonan I (RGI) polysaccharides from potato pulp has been reported (Thomassen et al., 2011b). The enzymatic process for such release applied a combination of fungally derived enzymes, including polygalacturonase (EC 3.2.1.15; Glycoside Hydrolase (GH) family 28) and pectin lyase (EC 4.2.2.10; Polysaccharide Lyase (PL) family 1), to degrade the unsubstituted regions of pectin homogalacturonan (HG), thus, releasing the more decorated regions, especially RGI, of pectin (Thomassen et al., 2011b). These pectin regions also appear to be abundant in sweet potato pulp (Arachchige et al., 2021; Liu et al., 2021).

Enzyme-assisted release of specific structures from sweet potato pulp polysaccharides requires detailed knowledge of the composition of the processing pulp material and careful selection of enzymes. The most abundant region of the sweet potato processing pulp pectin is indeed RGI, composed of a backbone of alternating α-1,4 linked galacturonic acid (GalA*p*) and α-1,2 linked rhamnose (Rha*p*), with galactose (Gal*p*)- and arabinose (Ara*f*)-rich sidechains. One likely sidechain is arabinogalactan (AG) Type I consisting of a β-1,4 linked Gal*p* backbone with α-1,3 and α-1,5 linked Ara*f* substitutions (Hinz et al., 2005). In addition, low levels of β-1,3 linked Gal*p* have been reported in potato, soy, onion, and citrus fruit, corresponding to about 0.5% of the β-1,4 linked Gal*p* (Hinz et al., 2005). The branched RGI regions of pectin are usually not degraded by the classical industrially available pectinases, which mainly act on the smooth HG regions of the pectin. Meanwhile, studies of human colonic *Bacteroides* species, where the metabolism of poly saccharides, including RGI, is programmed by a series of genes encoding for several different enzymes in polysaccharide utilization loci (PULs), have underlined the large repertoire of CAZymes needed for RGI degradation (Luis et al., 2018). Accordingly, selective modification or degradation of RGI present in sweet potato pulp, i.e., modification of either the RGI backbone or its sidechains to obtain specific, defined structural moieties, requires identification and/or discovery of distinct enzymes.

*Penicillium sclerotigenum* is a well-known post-harvest plant pathogen of certain tubers (Kim et al., 2008). The taxonomical section *Penicillium* indeed harbors several other specialized species of post-harvest plant pathogens against pectin-rich crops e.g., apple and citrus fruits (Barrett et al., 2020b).

We hypothesized that this fungus would be able to secrete novel pectin-modifying enzymes capable of acting on sweet potato pulp. To test this hypothesis, we got the *P. sclerotigenum* genome sequenced and then examined whether certain enzymes would be induced when *P. sclerotigenum* was exposed to the pectinaceous polysaccharides of sweet potato tuber pulp. In order to study such induction of enzymes, we generated a de-starched complex pectin fraction rich in RGI branched with AG Type I. We then inoculated the fungus on this fraction (used as the sole carbon source) and used bioinformatics analysis of the carbohydrate-active enzyme genes in the *P. sclerotigenum* genome and explorative proteomics analysis of the CAZymes secretome to examine the enzymes expressed after four days of incubation. Here, we report both the genome sequence of *P. sclerotigenum* and a catalog of CAZymes being expressed by the fungus in response to the pectin-rich fraction. In addition, we describe the finding of one unclassified Glycoside Hydrolase (GH0) and five novel secretome proteins that were induced upon exposing *P. sclerotigenum* to a complex sweet potato pectin fraction as the sole carbon source.

## Materials and methods

Monosaccharide analysis. Monosaccharides were obtained by acid hydrolysis according to the process used by Sluiter *et al.* (2008) with a few modifications: All samples were lyophilized prior to hydrolysis. Insoluble substrates were treated in 72% sulfuric acid for 1 h at 30°C, followed by dilution to 4% sulfuric acid and autoclaving for 1 h at 121°C. Soluble substrates were dissolved directly in 4% sulfuric acid and autoclaved for 1 h at 121°C. All samples were cooled immediately after autoclaving and stored at 4°C until further use. Monosaccharides were measured using a Dionex HPAEC-PAD system equipped with a PA1 column and involving post-column NaOH addition, as described by Zeuner et al. (2018).

**FIGURE 1:**
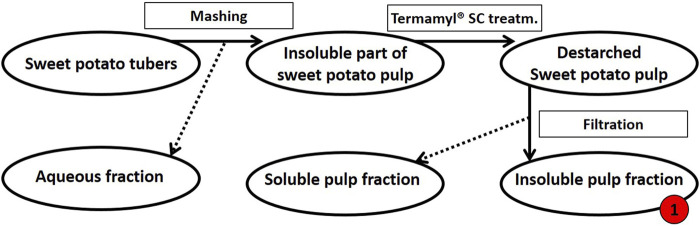
Schematic flow diagram of the mashing process of sweet potato tubers followed by de-starching. Processing of 500 g fresh sweet potato tubers resulted in a dry matter yield of 112 g insoluble pulp (this destarched insoluble fraction of pulp is called fraction 1, as indicated in red). The insoluble pulp fraction was treated further, see Figure 2.

Extraction of tuber polysaccharides. The sweet potato tubers (variant Yanshu 25) were purchased from the local market in Copenhagen, Denmark. As summarized in Figure 1, the sweet potatoes were then processed as follows: To obtain the destarched pulp, the tubers were washed prior to mashing in a Philips Juicer HR1865 with a built-in centrifuge and micro mesh filter to separate the liquid from the insoluble pulp. The insoluble pulp was washed with milli-Q^®^ water. For de-starching, the pulp was diluted in deionized water to 8% (w/w) dry matter and heated to 70°C before the addition of 0.2% (v/w dry matter) Termamyl^®^ SC (Novozymes A/S, Bagsværd, Denmark) and the pulp suspension was then allowed to react for 65 min (Thomassen and Meyer, 2010). The pulp was washed with milli-Q^®^ water and filtered using a 12-inch Porcelain Büchner Funnel to remove the solubilized starch sugars resulting in destarched sweet potato pulp (Figure 1).

**FIGURE 2:**
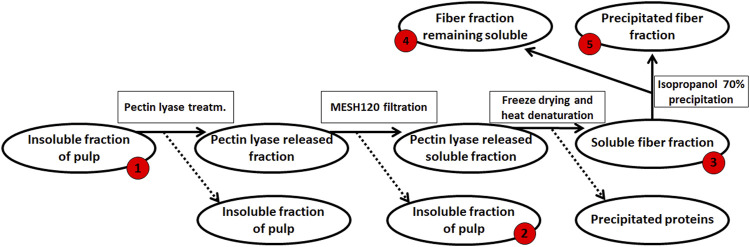
Enzymatic extraction of pectin-rich polysaccharides from de-starched sweet potato processing pulp. The red numbers indicate the fraction for which monosaccharide compositions were determined, from #‘s 1–5. The final extract (2.8 g) was resuspended in 200 ml ddH_2_O for further analysis.

The resulting de-starched processing pulp was then further processed to obtain a pectin-rich extract (steps summarized in Figure 2): The de-starched pulp was treated with the following fungal pulp-acting enzymes: Glycoside Hydrolase 3 (GH3, CAB75696.1) with β-glucosidase activity, EC 3.2.1.21 from *Aspergillus niger*. Glycoside Hydrolase 5 (subfamily 5 of GH5, AAC08587.1) with cellulase activity, EC 3.2.1.4 (this enzyme was a cloned enzyme originating from *A. aculeatus* and was supplied by Novozymes A/S (Bagsværd, Denmark), Glycoside Hydrolase 6 cellulose 1,4-β-Cellobiohydrolase (EC 3.2.1.91, CBHII, GH6, ABF50873.1) with non-reducing end activity (Gavlighi et al., 2013), and Pectin Lyase 1 (EC 4.2.2.10, subfamily 4 of PL1, EAA64674.1) (Thomassen et al., 2011a), with a preference toward linkages of the methylated moieties in the homogalacturonan backbone. The Cellobiohydrolase and the Pectin Lyase were cloned from *A. nidulans* FGSC A4 obtained from the Fungal Genetic Stock Center (Bauer et al., 2006) and produced recombinantly in *Pichia pastoris* in-house by high cell density fermentation as described previously (Michalak et al., 2012). The individual enzymes were added to the pulp at a concentration of 1% (w/w dry matter) and incubated for 20 h at 40°C. The remaining insoluble pulp was removed using a mesh sieve allowing a particle size of 0.09 mm and then discarded (Figure 2). The resulting liquid was freeze-dried to concentrate the polysaccharide, re-dissolved in milli-Q^®^ water, and solid–liquid separation was then achieved by centrifugation at 5,000 *g* for 10 min. The polysaccharide solution was heated to 95°C for 2 min, and the resulting precipitate was discarded. The large molecular weight polysaccharides were separated from the soluble mono- and oligo-saccharides by 70% cold isopropanol precipitation (Zha et al., 2014), and the extracted polysaccharide pellet was finally obtained by centrifugation at 10,000 *g* for 10 min; the supernatant, containing the mono- and oligo-saccharides, was discarded. The process resulted in 2.8 g DM pectin-rich extract (Figure 2). The monosaccharide composition was determined for the fractions obtained at the different stages in the extraction process; the stages were numbered as steps 1–5 (Figure 2). A negative control was included in the extraction accomplished in a similar manner but without the addition of pulp-acting enzymes.

Glycosidic linkage analysis by methylation. Glycosidic linkages present in the complex pectin-rich fraction were determined using methylation analyses (after carboxyl reduction of uronic acid residues) to form the partially methylated alditol acetates, which were then separated, identified, and quantified (as mol%) using the GC–MS according to the method outlined by Pettolino et al., 2012). These data were then used to propose the polysaccharides present in the fraction based on our knowledge of plant cell wall polysaccharides (Pettolino et al., 2012).

Extraction, sequencing, and assembly of fungal DNA. The fungus *P. sclerotigenum* IBT 15061 was obtained from our in-house fungal strain collection (IBT Culture Collection of Fungi, World Federation of Culture Collections (WFCC) no. 758). Spores from the culture collection were defrosted from storage at −80°C and inoculated onto Potato Dextrose Agar at 30°C. Fresh spores were harvested after ten days as follows: sterile water was added on top of the spore-covered mycelium in the agar plate, and the spores were then gently released with a sterile inoculation loop. 50 ml Malt Extract Broth medium were then inoculated with the *P. sclerotigenum* spore suspension (100,000 spores/mL medium) and incubated at 30°C for four days. The fungal biomass was separated from the medium by centrifugation at 10,000 *g* for 10 min and lyophilized. DNA extraction was performed using the standard protocol for the FastDNA Spin kit for Soil (MP Biomedicals, United States ) with the following modifications: 50 mg of the lyophilized fungal biomass was suspended in 500 µl sterile water; this suspension was denoted as the sample. Then, the 500 µl sample, 480 µl Sodium Phosphate Buffer, and 120 µl of MT Lysis Buffer were added to a Lysing Matrix E tube. Bead beating was performed at 6 m/s for 4 × 40 s (Albertsen et al., 2015). Gel electrophoresis using Tapestation 2,200 and Genomic DNA screentapes (Agilent, United States ) were used to validate the product size and purity of a subset of DNA extracts. DNA concentration was measured using a Qubit dsDNA HS/BR Assay kit (Thermo Fisher Scientific, United States ). Sequencing libraries were prepared using the NEB Next Ultra II DNA library prep kit for Illumina (New England Biolabs, United States ) following the manufacturer’s protocol. The sequencing libraries were pooled in equimolar concentrations and diluted to 4 nM. The samples were paired-end sequenced (2 × 300 bp) on a MiSeq (Illumina, United States ) using a MiSeq Reagent kit v3, with a coverage of at least 150 × (600 cycles) (Illumina, United States ) following the standard guidelines for preparing and loading samples on the MiSeq. The sequence reads were trimmed for adaptors using cutadapt (v. 1.16). The trimmed reads were assembled using megahit (v. 1.1.3 [li2016megahit]). The reads were mapped back to the assembly using minimap2 (v. 2.12-r827 (Li, 2018)) to generate assembly coverage files. The genome assembly has been deposited in the European Nucleotide Archive under code PRJEB50413.

*Ab initio* gene prediction was carried out with WebAUGUSTUS (Hoff and Stanke, 2013) using the reference proteome of *P. rubens* Wisconsin 54–1,255 as input. HAMAP (Pedruzzi et al., 2015) was used for functional annotation of proteins setting the annotation NCBI taxid to 254,878 (*P. chrysogenum* species complex). Data analysis was carried out in R (v. 3.5.1) using the R-studio environment and the mmgenome2 package (v. 2.0.12 (Karst et al., 2016)).

Preparation of polysaccharide growth media. A salt solution with a nitrogen source and minerals (4.5 g/L NH_4_NO3, 2.0 g/L KH_2_PO_4_, 0.15 g/LMgSO_4_·7H_2_O) and trace elements (0.05 mg/L FeSO_4_, 0.014 mg/L ZnSO_4_.7H_2_O, 0.016 mg/L MnSO_4,_ and 0.016 mg/L CoCl_2_) was autoclaved (Huang et al., 2014). The isopropanol sterilized extracted pectinaceous polysaccharide was added to a growth media in 20 g/L as the sole carbon source. A negative control was included with the addition of 20 g/L Glc instead of the extracted polysaccharides.

Fungal growth studies on sweet potato–derived pectin. The strain *P. sclerotigenum* (IBT 15061) was inoculated at 30°C on Potato Dextrose Agar for ten days before harvesting the spores as described earlier. Then, the growth medium containing the extracted sweet potato–derived pectin-rich fraction growth media (20 g/L, as described earlier, in 15 ml) was inoculated with a *P. sclerotigenum* spore suspension (100,000 spores/ml media) and incubated for four days at 30°C. The fungal biomass was separated from the media by centrifugation at 10,000 *g* for 10 min, and the supernatant was subjected to proteomics analysis, as described next.

Sample preparation for proteomics analysis. The supernatant for fungal incubation was treated with trichloroacetic acid (TFA) to a final concentration of 10% (v/w) and stored overnight at −20°C (Niu et al., 2018). The precipitated proteins were centrifuged (10,000*g* for 30 min) into a pellet and then washed three times by repeated addition of ice-cold acetone and centrifugation at 14,000 *g* for 5 min. The washed protein pellet was air dried prior to trypsin digestion and MS-based proteomics (see in the following section). For the determination of the intracellular proteins, the pulverized fungal biomass was re-suspended in water and centrifuged (10,000 *g* for 10 min) prior to TFA treatment and the sequential washing in ice-cold acetone.

Exo-proteomic analysis. MS-based proteomics analyses were carried out as described previously (Pilgaard et al., 2019). In brief, aliquots (10 μg of protein) of the precipitated, washed, and dried protein samples were diluted in 10% acetonitrile and 50 mM HEPES buffer pH 8.5 and digested with LysC (Wako, Osaka, Japan) for 4 h at 37°C and then by trypsin (Sigma-Aldrich, St. Louise, MI, US) overnight at 37°C. The digests were desalted (C18 filters (Thermo Fisher Scientific, Rockford, United States ) and then subjected to LC-MS/MS on an EASY nLC-1200 liquid chromatography system (Thermo Scientific) coupled to a Q-Exactive HF-X Orbitrap LC-MS. Peptides were separated on a 15 cm EASY-spray column (2 µm C18 particles and inner diameter 75 μm; Thermo Scientific) by elution by a gradient of solvents A (0.1% formic acid) and B (80% acetonitrile, 0.1% formic acid) for 70 min. Full scans were acquired in the Orbitrap with a resolution of 60,000, automatic gain control set at 3 × 10^5^ and a maximum injection time of 20 ms with 300–1750 m/z as the set range. Protein identification was performed using the open-source software MaxQuant (1.6.3.4) (Tyanova et al., 2016a) and Perseus (Tyanova et al., 2016b) *via* https://maxquant.net/and https://maxquant.net/perseus/. A hit was only considered if present in at least two out of three biological replicates. The proteins were annotated using the CUPP.INFO online platform with default settings (Barrett et al., 2020a).

## Results

### Composition of pectinaceous polysaccharide fraction (extract)

Monosaccharide compositions at different stages of extraction were obtained to better understand the biomass (Table 1). As expected, the monosaccharide composition at the different stages of destarching and further enzymatic extraction (Figure 1, 2) showed a decrease in the relative glucose content, whereas the relative content of monosaccharides associated with pectin and particularly with RGI, i.e., arabinose, rhamnose, galactose, and galacturonic acid, increased (Table 1). The mannose present is considered to be a contamination from the *Pichia pastoris* expressed enzymes used for the extraction (Gidley and Reid, 2006; Cuskin et al., 2015).

**TABLE 1:** Monosaccharide composition (mg/20 g DW) in dehydrated de-starched pulp determined at different stages of extraction, described in Figure 1 and notably in Figure 2, using enzymatic extraction and 70% isopropanol (IPA) treatment. Values are averages of three independent measurements ±s.d.

	Fuc	Ara	Rha	Gal	Glc	Xyl	Man	GalA	GlcA	Sum
No.1 Destarched pulp	10 ± 1	534 ± 16	267 ± 20	993 ± 26	9,435 ± 595	174 ± 22	45 ± 9	1,490 ± 161	22 ± 5	12,972
No.2 Remaining pulp	4.4 ± 0.6	130 ± 13	64 ± 14	88 ± 12	5616 ± 306	137 ± 38	60 ± 10	502 ± 98	13 ± 9	6616
No.3 Solubilized pulp	4.3 ± 0.3	405 ± 8	137 ± 8	790 ± 2	3227 ± 7	66 ± 3	347 ± 13	949 ± 12	8.8 ± 0.6	5934
No.4 IPA solubles	0.6 ± 0.3	220 ± 11	9.5 ± 9.7	396 ± 22	3135 ± 26	40 ± 2	25 ± 10	251 ± 73	1.7 ± 0.4	4078
No.5 IPA precipitate	3.3 ± 0.0	214 ± 5	102 ± 0	425 ± 12	265 ± 53	15 ± 2	189 ± 1	733 ± 9	5.1 ± 0.1	1951

The results of the linkage analysis outlined the constituent carbohydrate structural elements. The linkage analysis of the extract (the 70% IPA precipitate, extract no. 5), thus, substantiated that the extract mainly comprised RGI with AG Type I branches that were most probably attached to the Rha*p* in the RGI backbone (Table 2). The Ara*f* was found as terminal residues, meaning that there was no evidence of linear or branched arabinan. Some of the t-Ara*f* might be attached at the branching points of the AG Type I galactan sidechain, which would be consistent with current interpretations of RGI-AG Type I data. In the RGI backbone, five out of six Rha*p* have a side-chain likely to be AG of Type I. None of the GalA*p* is substituted, excluding the possibility of the presence of xylogalacturonan. Similarly, the t-Xyl*p* cannot be present in xyloglucan without 1,4,6-Glc*p* present. Fuc*p* has been reported as a terminal sugar in AG Type I attached to the C-4 (O’Neill and York, 2003).

**TABLE 2:** Carbohydrate linkages of each of the monosaccharides were determined by linkage analysis. The values listed are the molar percentage, and the mg is reported as dehydrated mass. AG–Arabinogalactan, HG–Homogalacturonan, and RGI–Rhamnogalacturonan I. Values in bold are summarizing the values of the polysaccharide type above.

		Derivative linkage	Content [Mol%]	Pectin extract [mg pr. 20 gDW]
**Pectic Polysaccharides**	**AG - Type I**	1,4-Gal*p*	13.0	254.0
		1,2,4-Gal*p*	0.9	17.5
		t-Gal*p*	7.8	152.4
		t-Ara*f*	5.2	121.8
		t-Fuc*p*	0.2	3.3
		**Total AG - Type I**	**27.0**	**548.9**
	**AG - Type II**	1,3-Gal*p*	0.1	1.1
		**Total AG - Type II**	**0.1**	**1.1**
	**HG**	1,4-GalA*p*	30.1	512.3
		t-GalA*p*	5.5	100.4
		**Total HG**	**35.7**	**612.7**
	**RGI**	1,4-GalA*p*	4.7	120.2
		1,2-Rha*p*	0.9	18.3
		1,2,4-Rha*p*	3.9	83.3
		**Total RGI**	**9.5**	**221.9**
		**Total Pectic Polysaccharides**	**72.2**	**1,384.6**
**Xylan**		1,4-Xyl*p*	0.2	3.7
		**Total Xylan**	**0.2**	**3.7**
**Cellulose**		1,4-Glc*p*	6.6	128.4
		**Total Cellulose**	**6.6**	**173.1**
**Yeast β-glucan**		1,6-Glc*p*	1.6	32.2
		1,3,4-Glc*p*	0.9	17.8
		1,2,6-Glc*p*	0.9	18.1
		t-Glc*p*	1.8	42.3
		**Total Yeast β-glucan**	**4.1**	**91.5**
**Yeast Mannan**		1,2-Man*p*	2.1	41.4
		1,2,3-Man*p*	0.1	2.3
		1,2,6-Man*p*	0.1	2.2
		1,3,6-Man*p*	0.2	4.1
		t-Man*p*	7.1	139.3
		**Total Yeast Mannan**	**9.7**	**189.2**
**Undefined**		t-Ara*p*	3.9	92.3
		t-Xyl*p*	0.4	8.4
		1,2-Xyl*p*	0.1	2.9
		t-Glc*p*	1.1	25.8
		**Total Undefined**	**6.7**	**148.3**
		**Total**	**100.0**	**1950.9**

Interpretation of the monosaccharide composition in the context of known compositions of pectin could reveal relevant insight into the composition of the sweet potato tuber polysaccharides. The Rha*p* is likely to be a part of the RGI backbone of pectin in a 1:1 ratio with GalA*p*. Pectin might have side-chains rich in Ara*f* and Gal*p*. Ara*f* and Gal*p* were, thus. found to be abundant in the polysaccharide extract with 2.4 and 11 times the amount in moles relative to that of Rha*p*, respectively. Such a composition has been reported to be present in AG substituted on an RGI backbone across a broad span of taxonomies (Ndeh et al., 2017). Additionally, the remaining GalA*p* has been reported in other plants primarily in the form of HG and combined, the RGI, AG Type I and HG constituted 76% of monosaccharides in the polysaccharide extract (No. 5 in Table 1).

The pectin likely consists of a range of different sizes and structures. To further elaborate on the possible association between the monosaccharides, the polysaccharide extract (fraction no. 5,) was further separated by 60% IPA precipitation (Table 3).

**TABLE 3:** Monosaccharide composition of extracted polysaccharides (No. 5. IPA precipitate, Table 1) and the soluble (solubles 2) and insoluble fraction (precipitate 2) results from an additional treatment with 60% IPA (IPA) on the obtained pectin-rich polysaccharide fraction. The weight of the monosaccharides is provided if they were dehydrated in mg/20 g DW destarched pulp. The values are the average of three independent measurements, including the standard deviation of the three.

	Fuc	Ara	Rha	Gal	Glc	Xyl	Man	GalA	GlcA	Protein
No.5 Precipitate	3.3 ± 0.0	214 ± 5	102 ± 0	425 ± 12	265 ± 53	15 ± 2	189 ± 1	733 ± 9	5.1 ± 0.1	626
Further fractionation										
No.5 Solubles 2	0.0 ± 0.0	147 ± 1	8.1 ± 0.1	239 ± 4	229 ± 8	7.5 ± 0.7	17 ± 3	341 ± 5	0.7 ± 0.3	232
No.5 Precipitate 2	3.2 ± 0.5	65 ± 2	92 ± 5	180 ± 1	32 ± 3	7.4 ± 1.4	168 ± 3	380 ± 74	4.3 ± 2.6	350

In the 60% IPA, the soluble fraction had a low content of Rha*p* relative to the Ara*f* and Gal*p* content. This may indicate that RGI is present with large galactan-rich side chains. In the 60% isopropanol precipitated fraction of the polysaccharide extract, the majority of the Rha*p* was found, along with low content of Gal*p* and Ara*f* compared to the soluble fraction. The backbone of RGI often has Ara*f* and Gal*p*-rich sidechains on the Rha*p* residues.

To summarize, from the monosaccharide compositions presented in Table 3, the polysaccharide extract contains GalA*p* in the 60% IPA soluble and precipitate fraction. The 60% IPA soluble fraction generally contains polysaccharides of shorter molecular weight, whereas the precipitate generally is of larger molecular weight (Zha et al., 2014). This result indicates that both the fractions contain mainly pectin-associated polysaccharides.

A large number of proteins (626 mg/20 g DW destarched pulp) was present along with the polysaccharides in the pectin-rich fraction (Table 1). MS-based proteomic analysis of the polysaccharide extract (No. 5) detected that 50% of the proteins were sweet potato storage proteins known as sporamin (Shewry, 2003). Second, two proteins predicted to be heme peroxidases accounted for 27% of the proteins in the extract. The pectin lyase added for enzymatic extraction of the polysaccharides was also observed in the extract and accounted for 12% of the total protein content. The added CBHII was also observed and accounted for about 1% of the proteins, whereas those remaining were less abundant plant proteins. This quantity of *Pichia* expressed enzymes is enough to account for the increase of mannose observed in between the monosaccharide composition analysis of the destarched pulp (fraction No. 1) and the enzymatically treated pulp (fraction No. 3).

### Fungal digestion of pectin-rich fraction from sweet potato tubers

The extracted polysaccharide was analyzed before and after four days of incubation with *P. sclerotigenum* (Table 4). From the compositions, the side-chains of the RGI appeared to be degraded, whereas the monosaccharides from the RGI backbone, Rha and GalA, appeared to remain in a ratio of about 1:1.

**TABLE 4:** Monosaccharide composition analysis of the polysaccharide extract (fraction No. 5) before and after four days of inoculation with *P. sclerotigenum*. The values are the average of three independent measurements ±s.d.

	Fuc	Ara	Rha	Gal	Glc	Xyl	Man	GalA	GlcA	Protein
No.5 Precipitate	3.3 ± 0.0	214 ± 5	102 ± 0	425 ± 12	265 ± 53	15 ± 2	189 ± 1	733 ± 9	5.1 ± 0.1	626
No.5 Post incubation	3.3 ± 0.0	12 ± 2	78 ± 13	22 ± 2	20 ± 5	6.4 ± 0.3	173 ± 24	88 ± 17	3.3 ± 0.1	141
Fungal consumption	0%	94%	23%	95%	92%	57%	9%	88%	35%	79%

#### Genomic annotation of *Penicillium sclerotigenum*

The genomic DNA was extracted from *P. sclerotigenum* and full genome sequenced to obtain a coverage of 151.7 × with 8′398′647 reads*.* The length of the genome assembly was 32 Mb and, in total, 9,085 proteins were predicted, of which 376 were predicted to be carbohydrate-active enzymes (CAZymes): the *ab initio* protein prediction was annotated using CUPP (Barrett and Lange, 2019; Barrett et al., 2020a) and the dbCAN2 pipeline, including Hotpep, dbCAN, and Diamond (Zhang et al., 2018). To obtain an overview, the CAZymes annotated by either dbCAN2 or CUPP are included in Table 5. The families highlighted by underline in Table 5 are known to contain at least one member associated with pectin modification (in this analysis, the possible sidechains of pectin are also considered part of pectin, e.g., arabinogalactans). Within each of the CAZy families, multiple functions may be present, as in the case of Glycoside Hydrolases family 2 (GH2). For example, among the six CAZyme proteins belonging to GH2 identified in *P. sclerotigenum* (Table 5), two were predicted to be *ß*-galactosidases and counted as probably acting on pectic galactans, whereas the remaining four proteins included non-pectin targets, i.e., two were β-mannosidases, one was an exo-β-glucosaminidase, and the last was of unknown function.

**TABLE 5:** CAZyme inventory consisting of 376 CAZymes encoded in the genome of *P. sclerotigenum* in their respective CAZy families. The CAZy families indicated by red font are families with at least some members known to be associated with pectin degradation. AA**–**Auxiliary Activities, CE**–**Carbohydrate Esterases, GH**–**Glycoside Hydrolases, GT–Glycosyl Transferases, and PL**–**Polysaccharide Lyases.

**AA**	**CE**	**GH**					**GT**			**PL**
AA1 8	CE0 1	GH0 2	GH15 3	GH35 3	GH63 1	GH92 4	GT0 1	GT25 3	GT57 2	PL1 3
AA3 25	CE3 1	GH1 3	GH16 15	GH36 2	GH64 1	GH93 1	GT1 7	GT28 1	GT58 1	PL3 1
AA4 1	CE4 2	GH2 6	GH17 5	GH37 1	GH65 1	GH95 1	GT2 18	GT31 6	GT59 1	PL4 2
AA5 2	CE5 1	GH3 15	GH18 10	GH38 1	GH67 1	GH105 1	GT3 1	GT32 7	GT62 3	PL20 1
AA6 1	CE8 3	GH5 12	GH20 2	GH43 8	GH71 8	GH114 2	GT4 8	GT33 1	GT66 1	
AA7 7	CE9 1	GH6 1	GH25 1	GH47 7	GH72 6	GH125 1	GT8 4	GT34 3	GT69 2	
AA9 4	CE12 2	GH7 2	GH27 2	GH51 2	GH75 1	GH128 1	GT15 3	GT35 1	GT71 3	
AA11 2	CE16 1	GH10 3	GH28 9	GH53 1	GH76 8	GH131 1	GT20 5	GT39 3	GT76 1	
AA13 2		GH11 1	GH30 1	GH54 1	GH78 4	GH132 2	GT21 1	GT41 1	GT90 4	
		GH12 2	GH31 9	GH55 3	GH79 2	GH135 2	GT22 4	GT48 1		
		GH13 16	GH32 4	GH62 1	GH81 2	GH154 1	GT24 1	GT50 1		

The members of the CAZy family GH28 may all be associated with pectin degradation but act on distinct linkages. The encoded GH28s of *P. sclerotigenum* were predicted to contain one xylogalacturonanase, two exo-polygalacturonosidases, two polygalacturonases, one rhamnogalacturonase, one rhamnogalacturonan α-1,2-galacturonohydrolase (Table 6), and two enzymes had unknown function. The GH35 may have members acting on Gal substitutions present in arabinogalactan for example. One of the CAZymes is of unknown molecular function, whereas one was annotated as a β-galactosidase (Table 6). *P. sclerotigenum* was also predicted to encode eight members of the polyspecific family GH43 (Table 5); three of these were annotated to be endo-1,5-α-L-arabinanases, one as an α-l-arabinofuranosidase/β-xylosidase, and several have no known function (Mewis et al., 2016; Helbert et al., 2019).

**TABLE 6:** Exo-proteome results of *P. sclerotigenum* and two studies of pectin-associated CAZymes found in *A. niger* strain NRRL3 with proteomics and transcriptomics data from various combinatory regulator knock-out studies (Kowalczyk et al., 2017) or regulator overexpression studies (Alazi et al., 2018). The CUPP groups and the accession number of at least one member of the group, if any. The asterisk indicates that multiple CAZymes are found in the respective CUPP group for the respective strain.

A. niger strain NRRL3 proteomics & transcriptomics by regulatory alterations									
P. sclerotigenum			JGI# Acc	Proteomics	Transcript	Fold change by transcriptomics			
CUPP Gr	Gene ID	wt - Fold increase		Fold change OEgaaR/ref	Abundance OEgaaR	OEgaaR/ref on fructose	ref/ΔrhaR|ΔaraR |ΔgaaR on pectin	ref/ΔrhaR on rhamnose	CUPP annotation
CE8:0	PenS1340		-		-				—
CE8:3	PenS5292		7470	++	58.0	++	++		Pectin methylesterase
CE8:6	-		8325	+++	24.7	+++	+++		Pectin methylesterase
CE8:16	PenS2947	+++	5252	+++	17.5	++	+++		Pectin methylesterase
CE12:1	PenS314	+++	169		0.8		+++	++	Pectin acetylesterase
CE12:4	PenS505	+++	7501	+++	1.7		+	++	—
CE16:0	PenS7186	+++	—		—				Acetylesterase
CE16:1	—		4916		0.4	+	++		Acetylesterase
CE16:2	—		6379*		24.5	+++	++		Acetylesterase
GH2:2	—		1731		0.0				β-glucuronidase
GH2:6	PenS6903		—		—				β-galactosidase
GH2:20	PenS7903		—		—				β-galactosidase
GH2:31	PenS6524	+++	6203		0.2				—
GH5:60	—		8701		0.6		++		Galactan endo-1,6-β-galactosidase
GH28:0	—	.	—		—				—
GH28:1	PenS8679*	.	2835*	+++	479.3	+++	+		Endo-polygalacturonase
GH28:3	PenS3966		9126*		1.3		+		Rhamnogalacturonase
GH28:6	PenS5291		7469	+++	4.8	++	++		Xylogalacturonan hydrolase
GH28:10	PenS6441	+++	3144*	+++	65.8	++	+++		exo-polygalacturonase
GH28:15	PenS1513	.	10559*	+++	16.9	+++	+++		Rhamnogalacturonan α-1,2-galacturonohydrolase
GH28:16	—		8631		0.2	+++	+		α-L-rhamnosidase
GH28:17	—		5260	+++	45.1	++	++		exo-polygalacturonase
GH28:18	PenS3938	+++	8281	+++	14.3	+++	+++		exo-polygalacturonase
GH28:25	—		—		—				—
GH28:36	PenS1632		4303		0.0				—
GH35:0	PenS6735	.	11738*		1.4	.	+++	++	—
GH35:2	PenS8981*	++	2630*		33.5	++	++		β-galactosidase
GH43:0	PenS2088		—		—				—
GH43:1	PenS588	++	—		—				β-xylosidase & α-L-arabinofuranosidase
GH43:25	PenS7824	++	92*		0.0		+++		endo-α-1,5-L-arabinanase
GH43:26	PenS345*		7094*		51.0		+		endo-α-1,5-L-arabinanase
GH43:50	PenS5768		6244		22.9	++	++		GH43 subfamily 13 - Unknown
GH43:79	—		11773		1.4		++		exo-β-1,3-galactanase
GH43:108	PenS7726		4608		5.4	+	+		GH43 subfamily 30-Unknown
GH43:109	PenS100	+++	3855		0.1		+		GH43 subfamily 30-Unknown
GH43:121	—		9890		0.4				GH43 subfamily 34-Unknown
GH51:2	—		10865		10.3	++	+++		α-L-arabinofuranosidase
GH51:4	PenS2692*		6387*	+++	1.8	.	++		α-L-arabinofuranosidase
GH53:1	PenS7308	+++	10643	+++	28.5	++	++		endo-β-1,4-galactanase
GH54:1	PenS3117		3768	+	10.1	++	+++		β-xylosidase & α-arabinofuranosidase
GH78:0	—		6304		1.3			++	—
GH78:1	—		7520		1.7			++	α-L-rhamnosidase
GH78:5	—		2162		8.3		.		α-L-rhamnosidase
GH78:6	PenS1514		10558		17.5	++	+++		α-L-rhamnosidase/rhamnogalacturonan α-L-rhamnohydrolase\
GH78:22	—		4245		0.0			+++	—
GH78:24	PenS1787*		-		—				—
GH78:25	—		3279		0.9		+	++	—
GH78:29	PenS1788		11451*		0.0		.		—
GH79:4	—		5305		2.7	++	+++	++	β-glucuronidase
GH79:7	PenS5991*	++	730	+++	2.9	.	++		-
GH95:1	PenS5210		7382		2.1	++			1,2-α-L-fucosidase
GH95:2	—		7089		11.2	+	+		1,2-α-L-fucosidase
GH105:0	—		1038		0.3		++		—
GH105:2	PenS6656		839		4.0		.	++	—
GH142:3	—		1368		2.7	+	+++		—
PL1:0	—		9811		0.5	+++			
PL1:12	PenS970*	+++	965*	+++	332.9	+++	+		Pectin lyase
L1:22	PenS6638		6359		1.4	+			Pectate lyase
PL3:5	PenS8263		—		—				Pectin lyase
PL4:1	PenS2474		684		0.3	++	++		Rhamnogalacturonan endolyase
PL4:3	—		—		—				Rhamnogalacturonan endolyase
PL4:5	PenS1337		10115		11.2	++	++	+++	Rhamnogalacturonan endolyase
PL9:11	—		—		-				—
PL26:1	—		—		-				Rhamnogalacturonan exolyase
PL27:1	—		707		0.0			+++	L-rhamnose-α-1,4-D-glucuronate

The two members of GH51 found in *P. sclerotigenum* are both predicted to be α-L-arabinofuranosidases (Table 6). Furthermore, one member of GH53 was predicted to act as an arabinogalactan endo-β-1,4-galactanase associated with pectin, whereas the single members belonging to GH54 and GH62 have been predicted to have a dual function as an *ß*-xylosidase and α-l-arabinofuranosidase and as an arabinoxylan-specific arabinofuranosidases, respectively (Kaur et al., 2015).

Three of the four members of GH78 have an unknown function (PenS1787, PenS1788 (Table 6), and PenS5989, Supplementary Table S1), whereas the fourth one has either an α-l-rhamnosidase or rhamnogalacturonan α-l-rhamnohydrolase activity acting on pectin (PenS1514, Table 6). Interestingly, one of the GH78 CAZymes is located in the genome only a couple of hundred base pairs from a pectin-associated GH28 CAZyme (PenS1513, Table 6). The two genes are found in two different reading frames pointing away from each other, indicating that the genes are indeed two separate genes and not erroneously *ab initio* prediction dual domain proteins (data not shown). This observation may suggest that these two CAZymes are co-regulated and appear to be a functional pair of CAZymes that collaborate in the degradation of unsubstituted RGI from the non-reducing end (Hawkins, 2015). Further inspection of the individual CAZymes relevant for pectin degradation revealed that three enzymes had a CBM module adjacent to the catalytic domain, namely, the GH54 (PenS3117, Table 6), which had a C-terminal CBM42, and two of the GH78 domain proteins had an N-terminal CBM67 module.

Regarding the two proteins in GH79 (Table 5), corresponding to PenS5991, Table 6 and PenS1790, Supplementary Table S1), both enzymes have an unknown function, but based on the activities found amongst the members of this CAZy family, action on the GlcA*p* substitutions observed in RGII (Ndeh et al., 2017) and arabinogalactan (Cartmell et al., 2018) is plausible.

The fungal carbohydrate esterases (CE) in family 8 act on methyl esters in pectin, whereas CE12 and CE16 generally act on acetyl esters. The CAZymes belonging to Polysaccharide Lyases (PL) family 1 and 3 are pectin or pectate lyases (Table 6), whereas the two PL4 CAZymes, PenS1337 and PenS2474, were predicted to be rhamnogalacturonan endolyases (Table 6). The Glycosyl Transferases (GT) are usually intracellular enzymes which do not directly interact with pectin, thus, none are underlined in Table 5.

Even though a particular CAZyme is encoded in the genome, it does not mean that the protein is ever naturally expressed by the fungus. On the other hand, encoded proteins which are not already known as CAZymes may not be selected for characterization unless a potential association to the targeted substrate can be established. Such an association can be established through assessment of the fungal exo-proteome in the context of a target substrate, described further in the following section.

### Proteomics analysis of the *P. sclerotigenum* secretome after sweet potato pectin exposure

MS-based proteomics was conducted to elucidate which battery of known and currently unknown CAZymes that were induced by *P. sclerotigenum* in the presence of the pectinaceous sweet potato polysaccharide fraction (as sole carbon source). A similar MS-based approach has been used previously to identify the induction of certain plant cell wall polysaccharide degrading enzymes (Tsang et al., 2009; Alazi et al., 2018; Pilgaard et al., 2019). The label-free proteomics analysis revealed 1969 proteins which were observed to be present in or secreted by the fungus grown on either the polysaccharide extract or the glucose control.

A total of 68 proteins observed in the proteomics analysis are CAZymes containing signal-peptides; Furthermore, 94 signal-peptide containing non-CAZymes were observed in the proteomics analysis (and, thus, presumed to belong to the secretome), and an additional 46 were assigned as CAZymes but without a signal-peptide. 262 of the predicted genome-encoded CAZymes with or without signal-peptides were not observed in the proteomics analysis. In the present analysis, it cannot be excluded that some CAZymes without a signal-peptide are present because some of the fungal cells may have been disrupted, thus, leaking intracellular proteins during growth or during separation of the mycelium from the pectinaceous sweet potato substrate. For a protein to be considered present, the protein needs to be covered by at least two unique peptides, and the proteins must be detected in at least two of the three replicates (Ong and Mann, 2005; Tsang et al., 2009; López-Mondéjar et al., 2016; Alazi et al., 2018). The CAZymes were assigned to different substrate moieties based on their predicted molecular function. The CAZymes with known association to pectin were generally upregulated, whereas CAZymes associated to other polysaccharides such as xyloglucan, starch, and cellulose degradation were downregulated (Burton and Fincher, 2009). Interestingly, the three CAZymes involved in the degradation of the RGI backbone were observed in the exo-proteome but not at abundances greater than the glucose reference, see Figure 2.

In this study, abundance was compared in relation to the total abundance of proteins observed in the exo-proteomic analysis. In Figure 2, CAZymes associated to pectin-modification is heavily upregulated when the fungus is exposed to the pectin-rich fraction. Additionally, CAZymes involved in xyloglucan and starch are shown to be present in low quantity, amounting to about 0.5% when combined compared to those with known association toward pectin, which reached 11.3% combined. The fungal cell wall degradation–associated CAZymes were also abundant (approx 5.7%). The α-mannan-associated CAZymes were observed with an abundance of 0.6%, which was expected because a fraction of the enzymes expressed recombinantly in *P. pastoris* used for the extraction process, were still present in the extract.

### Abundant proteins of unknown function in the exo-proteome

**FIGURE 3:**
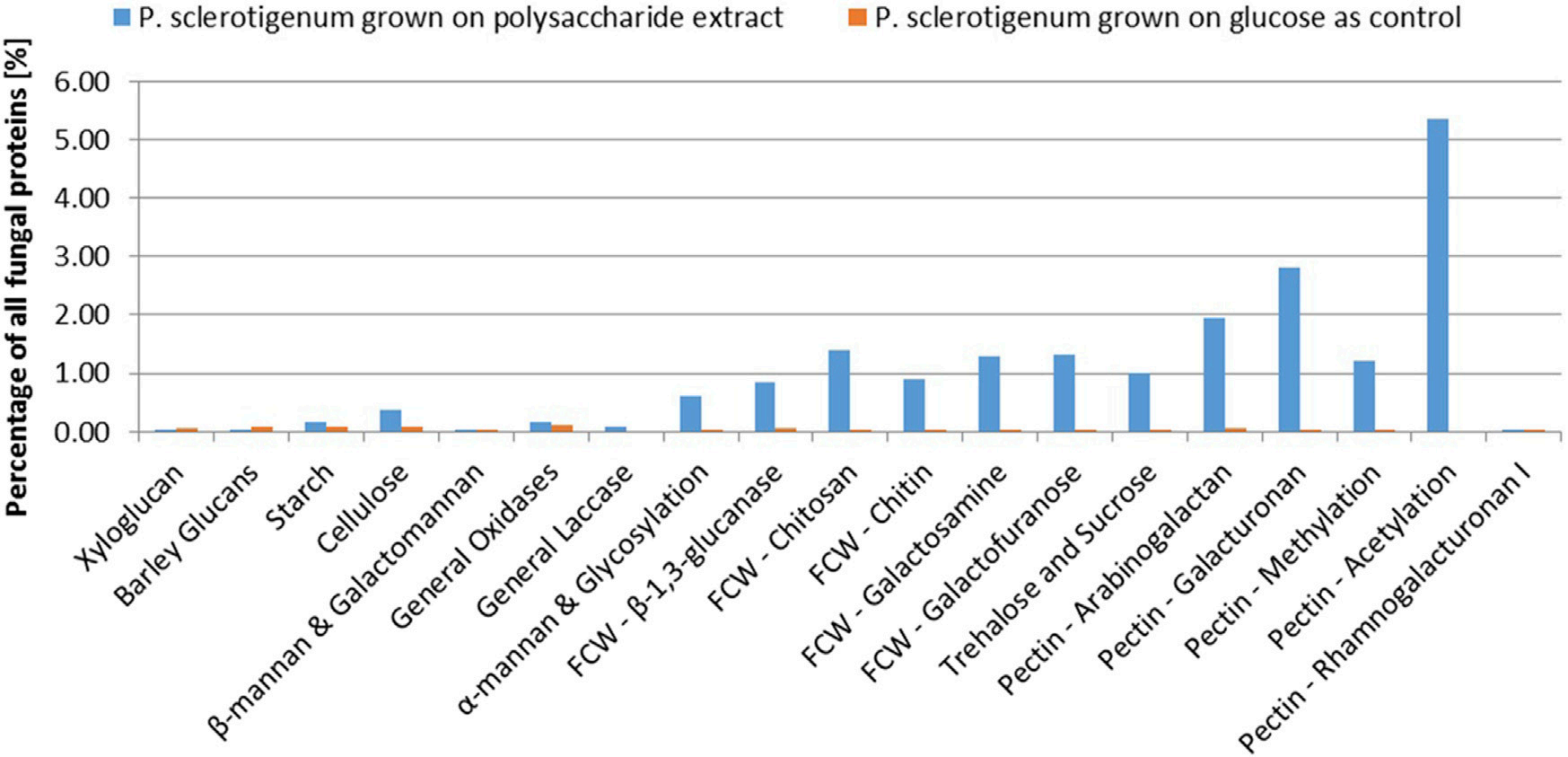
Abundance of each CAZyme category given as a percentage of the total protein content detected in the exo-proteome of *P. sclerotigenum*. The blue bars represent the abundance in the presence of the pectin-rich fraction, whereas the small orange bars to the right indicate the corresponding abundances in the reference grown on glucose. Prefix FCW indicates a suggested Fungal Cell Wall component target.

The CAZymes included in Figure 2 contributed to 20% of the total amount of extracellular proteins. The majority of the CAZymes could be functionally annotated by CUPP or annotated to a mono-specific CAZy family. Six signal-peptide–containing proteins out of the 94 not classified as CAZymes were observed in the exo-proteome and were responsible for 12% of the total protein content (Figure 3).

**FIGURE 4:**
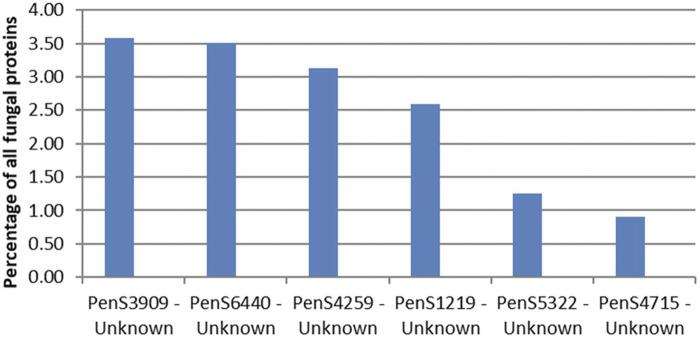
Protein abundances in the exo-proteome of six potentially new pectin active enzymes, all with predicted signal-peptide. The percentage level of each protein, shown as blue bars, is given as the abundance of the given protein relative to the total abundance of all proteins observed in the exo-proteome of *P. sclerotigenum*. PenSxx-numbers refer to protein accession numbers. The protein accession numbers match accession numbers of specific predicted proteins encoded by the genome of *P. sclerotigenum* (Supplementary Tables S1 and S2).

The most abundant unidentified protein from the proteomics analysis is the protein by the gene ID PenS3909 (Figure 4). This protein does not match any dbCAN, InterPro Scan, or CUPP models but structurally resembles the six-bladed beta-propeller, similar to that of GH93. The GH93 harbors characterized exo-α-L-1,5-arabinanases. The GH93 predicted in the genome of *P. sclerotigenum* did not appear to be present in the exo-proteome. The third signal-peptide–containing protein in Figure 3, gene ID PenS4259 (Figure 4), is predicted to be an unclassified Glycoside Hydrolase (GH0) by dbCAN. This GH0 protein was highly abundant, more so than most of the highly abundant enzymes acting on pectin and was amongst the most abundant proteins observed in the exo-proteome. The two other proteins, PenS6440 and PenS1219 (Figure 4), were assessed using InterPro Scan, which did not identify any protein family membership, but pfam gave an indication that they are an oxidoreductase and a protein predicted to have an association with neurotensin, respectively (Kim et al., 2018). The protein with gene ID PenS5322 (Figure 4) could not be predicted to any known protein family using InterPro Scan, pfam, dbCAN, and CUPP annotation tools.

### Catalogue of sweet potato pectin active CAZymes

CUPP groups are, in this context, used to interpret the result of the current *P. sclerotigenum* study to the results obtained previously from the related *A. niger* to build upon the previous work done in the area and directly link the results to a meaningful context.

In Table 6, the column “wt Fold increase” indicates the fold increase of the protein when *P. sclerotigenum* was grown on the pectin-rich sweet potato tuber fraction relative to the glucose reference, assessed through proteomics: three plusses indicate >100 times increase, two plusses signify >10 times increase, a single plus indicates >4 times increase, a dot is > 2 times, and nothing is below 2 times. Similarity, the plusses for *ΔaraR*, *ΔrhaR* and *ΔgaaR* indicate the increase of the particular genes in the reference strain relative to the strain with a gene knock-out of the regulatory genes involved in cell response to the presence of Ara, Rha or GalA. The *OEgaaR* refers to overexpression of the GalA regulatory gene, the fold increase of the mutant is presented relative to the wild-type strain. CUPP groups found in *A. niger* or *P. sclerotigenum* with an association to pectin are included, along with families having a general association of pectin.

**FIGURE 5:**
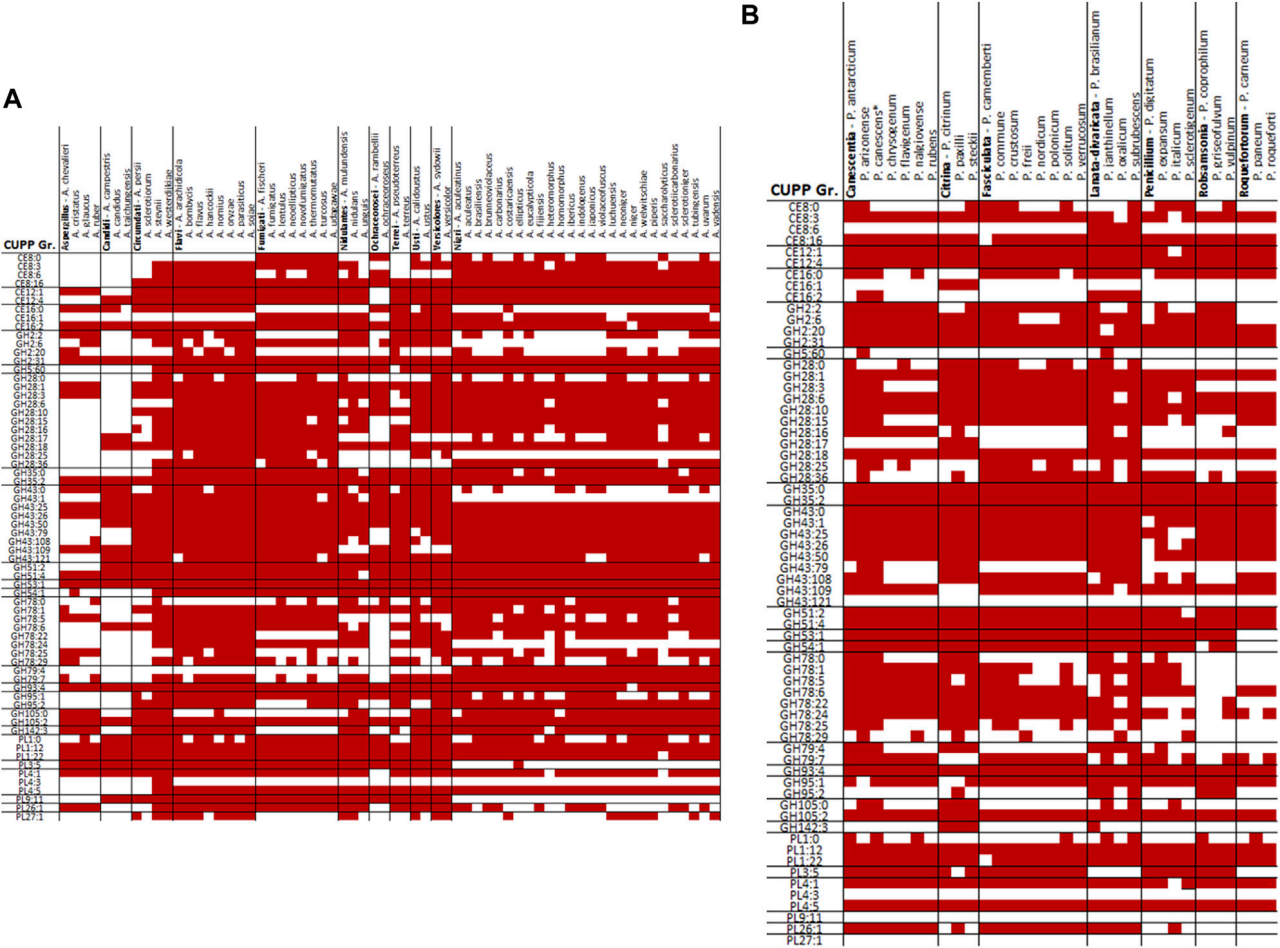
Overview of CUPP groups found in individual species of *Aspergillus* and *Penicillium* in red: **(A)** *Aspergillus* spp. and **(B)** *Penicillium* spp.

When comparing the CUPP groups predicted from *P. sclerotigenum* with those found in *Aspergillus* spp., including *A. niger* (Figure 5A) and in other *Penicillium* spp. (Figure 5B), a number of similarities and differences emerged. Several different CUPP groups were found within GH5, of which only CUPP group GH5:60 was associated to pectin in the proteomics study with *A. niger* strain ATCC1015 (Tsang et al., 2009). By inspection of the supplementary material of Kowalczyk *et al.*, it was possible to also see the regulatory response, particularly when the *araR* was knocked out (Kowalczyk et al., 2017). Furthermore, both *A. niger* and *P. sclerotigenum* have several CAZymes assigned to the same CUPP group within GH28 (Figures 5A,B), of which several appeared to be expressed by *P. sclerotigenum* when growing on pectin from sweet potato (Table 6).

The CAZyme belonging to CUPP group GH43:109 was observed in the exo-proteomics analysis of strain ATCC1015 when this strain was grown on pectin substrate, but also when the *A. niger* strain ATCC1015 was grown on the glucose control substrate, indicating a general purpose of this type of CAZyme. The enzyme was also observed in the OE*gaaR* strain but without an increase. In the *ΔgaaR* strain, the transcript level after 2 h of the CAZymes belonging to GH43:109 was more than 5 times lower than that of the reference, indicating an association to GalA-containing substrates. The GH43 subfamily 30 (Helbert et al., 2019) has recently had two members characterized as galactofuranosidase, but both characterized members are found in CUPP group GH43:106 and the two CAZymes found in this study belong to CUPP group GH43:108 and GH43:109.

The CAZy inventory of *A. niger,* including 21 GH28 CAZymes, spread across nine different CUPP groups. Seven of those GH28 CAZymes belong to CUPP group 1 of GH28, namely: NRRL_263, NRRL_2571, NRRL_2835, NRRL_4000, NRRL_5859, NRRL_6782, and NRRL_8805, all assigned as endo-polygalacturonases. Similarly, five different GH28 CAZymes belong to CUPP group 3, all assigned as endo-polygalacturonase, comprising: NRRL_953, NRRL_9126, NRRL_9450, NRRL_9623, and NRRL_11790. Also, two CAZymes belong to GH28 CUPP group 10, both assigned as exo-polygalacturonase active, namely, NRRL_3144 and NRRL_9810. Also, two CAZymes were found in CUPP group 15 of GH28, without a characterization available for any of the two, NRRL_2832 and NRRL_10559, but CUPP annotates them as rhamnogalacturonan galacturonohydrolase, EC number 3.2.1.173. The remaining five CAZymes of GH28 in *A. niger* belong to individual CUPP groups, some of which have distinct molecular functions.

The protein predicted to belong to CUPP group CE8:6 was found as a dual domain protein combined with the endo-polygalacturonase of CUPP group GH28 in all members of *Penicillium* section Lanata-divaricata and *Aspergillus* section Ochraceorosei as well as three members of *Aspergillus* section Fumigati and the species *A. nidulans*. An additional example is the two species *A. parasiticus* and *A. arachidicola* having a dual domain protein of CE12:1 with CUPP group GH28:16, exhibition pectin acetylesterase and alpha-l-rhamnosidase activity, respectively.

The two species *A. terreus* and *A. udagawae* had a CUPP group GH2:6 combined with CUPP group GH5:60, which acts as beta-galactosidase and galactan endo-1,6-beta-galactosidase, respectively. CUPP group GH28:16 is found combined with two pectin-associated CUPP groups, namely, PL3:5 and GH78:6 exhibiting alpha-l-rhamnosidase and pectin lyase activity. The PL3:5 combinations were found in two species of section Fumigati, namely, *A. lentulus* and *A. neoellipticus*, whereas the GH78:6 was found in *A. nidulan*, *A. calidoustus* and *Aspergillus* section Versicolores. The CUPP group GH43:25 exhibited arabinan endo-1,5-alpha-l-arabinosidase activity was found combined with the CUPP group PL9:11 assigned as a pectin lyase in *A. ochraceoroseus*. In *A. ustus*, *A. versicolor*, *A. lentulus,* and all members of the section *Aspergillus* sections Nidulantes and Terrei, a dual domain CAZyme, combining GH78:24 with GH79:7 was found. Since both CUPP groups harbor no characterized members, it is difficult to predict the likely natural substrate of these enzymes.

The majority of relevant CAZymes in *P. sclerotigenum* was also found in *A. niger* and appeared to be transcribed and translated consistently for the same CUPP groups. Additionally, 27 different CUPP groups with potential association to pectin degradation were identified in *Aspergillus* or *Penicillium* species other than *P. sclerotigenum* or *A. niger*. These were as follows: CE12:0, GH2:8, GH2:25, GH35:1, GH35:7, GH35:9, GH43:65, GH43:66, GH43:81, GH43:83, GH43:86, GH43:130, GH53:0, GH78:2, GH78:12, GH78:30, GH79:0, GH93:0, GH93:1, GH95:0, GH95:5, GH105:13, PL1:5, PL1:6, PL1:13, PL1:35, and PL3:0 (Figures 4A and B).

## Discussion

This study revealed that *P. sclerotigenum* encodes a large battery of CAZymes relevant for pectin degradation. Some of the *P. sclerotigenum* enzymes match the CUPP group category where a protein from *A. niger* also exists and is upregulated upon the presence of pectin. For example, it is known that the galacturonic acid moieties constituting the pectin backbone homogalacturonan may be heavily methylated at C6. Indeed, the CE8:16 protein (gene ID PenS2947, Supplementary Table S1) might catalyze the removal of the methylation of pectin, acting as a pectin methyl esterase, on sweet potato pectin (Table 6). With regard to acetyl esterases, the two enzymes from CE12 (PenS314 and PenS505), of which only PenS314 was annotated by CUPP to be an acetyl esterase (Table 6), and the enzyme from CE16, CUPP group CE16:0 (PenS7186, Table 6), appear to be highly important as they together constituted about 5% of the total protein content of the detected exo-proteome. Hence, although only one of these enzymes, namely CE12:1, PenS314, was predicted directly to be a pectin acetyl esterase, these findings strongly indicate the likely presence of heavy acetylation in the backbone of sweet potato HG alongside C6 methylations and emphasize the significance of deacetylation for depolymerization of the backbone. Treatment with the pectin methyl esterase and the three acetyl esterase might be a prerequisite for certain enzymes belonging to GH28 enzymes to act properly in the HG backbone. At the same time, the presence of multiple PL1 encoding genes in the *P. sclerotigenum* genome (Table 5) and the proteomic detection of a PL1 response for the fungal growth on the sweet potato pectin, notably the PL1:12 (PenS970) (Table 6), support that *P. sclerotigenum* uses several strategies to accomplish the degradation of complex substituted pectin. Taken together, these observations align well with genomics and enzyme data for other fungi. First, for example, for *Aspergillus* spp., it is known that those that efficiently degrade pectin may not only employ different enzymatic strategies simultaneously (Benoit et al., 2015), but may, in particular, have evolved genomes that grant significant “biological plasticity” (Zeuner et al., 2020). This biological plasticity enables the fungus to degrade an array of nearly similar complex structural carbohydrate elements, e.g., segments of pectin having different methoxylation and acetylation patterns, by means of encoding an array of similar types of enzymes having slightly different substrate preferences (Zeuner et al., 2020). It is generally known that fungi sense the surrounding carbohydrates and secrete proteins associated to the particular nearby biomasses (Tsang et al., 2009) and moreover tend to have a preferred order of which kind to consume first (Gong et al., 2015).

To obtain defined sub-structures of galactan-rich pectin on a short RGI backbone through tailored enzymatic extraction, the HG backbone needs to be decomposed. With the release of the methylation and acetylation, the HG regions flanking the RGI region are likely removed *via* the action of the two GH28 exo-polygalacturonase (PenS3938 and PenS6441, Table 6, g3938.1 and g6441. t1 Supplementary Table S1, S2). These two proteins belong to two different CUPP groups, thus, might have different behavior on the natural substrate. Additionally, the addition of the endo-polygalacturonase (PenS8679) may render the degradation of HG more efficient.

To further increase the ratio of galactan substitutions on the RGI backbone relative to arabinans, the two arabinan-associated CAZymes of GH43 (PenS588 and PenS7824) could be applied. A similar strategy was used for enzymatic removal of linear and branched arabinan to isolate a pure RGII pectin substructure (Ndeh et al., 2017). In case some of the arabinans still remain, it would be relevant to test the abundant protein with a predicted fold similar to the arabinan-associated CAZy family GH93 (PenS3909). Impure fractions could be treated with galactan-associated CAZymes to reduce the length of the galactan-substitutions, thus, reducing the molecular weight to enable separation. The galactan-associated CAZymes include two GH35 CAZymes (PenS6735 and PenS8981) and the GH53 (PenS7308).

In case some impurities exist in the final product, the molecular function of the abundant protein with affiliation to a GH0 family (PenS4259) could be tested to investigate the role this potential CAZyme might have on the natural substrate. Also, the CAZymes assigned to GH43:109 have been observed to be heavily abundant but with no characterized members. Similarly, the GH79 (PenS5991) is present in the exo-proteome but has not been characterized. The protein PenS4715 has similarities to the former CE10, which might have a yet unexplored involvement in the decomposition of plant cell wall materials. The CE10 has been deleted as the members appeared to be lipases rather than being active in carbohydrates. However, the CE10 enzyme might have a beneficial effect during the degradation of the pectin-rich sweet potato fraction. Also, the CAZyme of GH2 CUPP group 31 (PenS6524) have no known molecular function, and its potential application is yet to be established.

The CAZymes deemed relevant for tailored enzymatic extraction of sweet potato pectin substructures often have a counterpart in the studies performed for *A. niger* when grown on pectin. *A. niger* encodes two CAZymes that differ from the very abundant CE16 CAZyme observed in *P. sclerotigenum*. This could indicate that the pectin found in sweet potato is generally more acetylated than the pectin tested in the other studies or at least that acetyl is present and that the fungus is responding to its presence.

The presence of chitin and chitosan-associated CAZymes may be involved in the modification of the fungal cell wall, which has also been reported to be present regardless of the type of polysaccharides added (Tsang et al., 2009). It was reported that alterations of the fungal cell wall polysaccharides occur as part of the invasion strategy of certain fungi (Burton and Fincher, 2009; Fesel and Zuccaro, 2016). Furthermore, galactofuranose is also abundant in the fungal cells, for example, in *Aspergillus fumigatus,* and, therefore, the β-d-galactofuranosidase was categorized as a fungal cell wall acting enzyme (Lamarre et al., 2009). When the living plant senses the presence of certain fungal polysaccharides, it may initiate a defense mechanism to defeat the invader. Some plant pathogenic fungi, in response to such a recognition system, have developed counter measures involving the rearrangement of the fungal cell wall (Fesel and Zuccaro, 2016). Since components of the plant may be present in the polysaccharide extract, such a mechanism could be triggered, thus, explaining the upregulation of the non-pectin–associated CAZymes. The galactofuranosidase activity is potentially active on components in the fungal cell walls and not directly associated with pectin (Ramli et al., 1995). The two CUPP groups of this GH43 subfamily 30, however, still remain to be functionally characterized, but a possible association might be found since they have been reported to be co-regulated with genes directly associated to pectin (Kowalczyk et al., 2017). *A. niger* grown on six different carbon sources, including pectin, had hypothetical carboxylesterases present in the exo-proteome only when grown on pectin (Tsang et al., 2009). One of these is annotated as a CE10 by dbCAN, JGI gene ID 185301 (Tsang et al., 2009; Kowalczyk et al., 2017; Alazi et al., 2018). Based on the abundance of this protein in the current and former studies of similar CE10 proteins, we consider it likely that this particular protein has a desirable action on the pectin-rich sweet potato fraction.

The results of the study can be used in future research to link the individual CUPP groups to the action of the enzymes on particular substrates (down to action on specific linkages in particular substrates), thus, guiding the experimental characterization toward the most likely substrate. It appears that there is a need to expand certain CAZy families since the diversity is apparently not fully covered by the entries currently found in the open CAZy database. Four CUPP groups were based on the functional annotation potentially associated to pectin, but the representative CAZymes of *A. niger* and *P. sclerotigenum* were not observed as a result of the presence of pectin except one in *P. sclerotigenum*. Additionally, two GH2 CAZymes were found in the genome but not found in the exo-proteome of *P. sclerotigenum* and the functional annotation indicated molecular functions not relevant for pectin for CUPP groups GH2:19 and GH2:21. Furthermore, the studied regulators do generally not affect the GH2 CAZymes. The genome-encoded CAZyme assigned to GH62:1 was found in *A. niger* strain ATCC1015 in the presence of glucose and other non-pectin substrates, including locust bean gum (Tsang et al., 2009). This indicates that GH62 might not be a good candidate for identifying CAZymes active on pectin substrates and were not included in the overview.

*A. calidoustus* of section Usti feature CAZymes from 19 of the 31 CUPP groups not observed in *P. sclerotigenum* or *A. niger* which would make *A. calidoustus* a good place to look for functional diversity if the selection is restricted to *Aspergillus* or *Penicillium*. In principle, this analysis could go far beyond Ascomycota fungi. Such a grid could direct the biochemical characterizations toward the less explored groups of CAZymes and benefit the fields using the CAZymes for industrial applications such as targeted extraction of particular plant components (Holck et al., 2014).

## Conclusion

The genome of *P. sclerotigenum* was found to harbor genes for 376 known CAZymes, which could be annotated to belong to different GH, GT, PL, CE, and AA CAZy families. Twenty of the CAZymes families that the *P. sclerotigenum* enzyme proteins were annotated to are known to harbor enzymes involved in pectin degradation. By exposing the tuber pathogenic fungus *P. sclerotigenum* to a pectin-rich fraction from sweet potatoes prepared to resemble a complex pectin fraction present in sweet potato industrial starch processing pulp residues, we found induced expression of 68 secreted CAZymes. Minor structural variations may be found in sweet potato pectin of different sweet potato species or as a result of different levels of potato maturity or extraction or processing procedures. Nevertheless, the study is robust as it involved examination of a (new) fungal genome sequence using bioinformatics combined with Augustus annotation and CUPP subgrouping of CAZymes. The CUPP subgrouping relies upon the use of a newer algorithm for automatic classification (Barrett and Lange, 2019; Barrett et al., 2020a), which is completely reproducible for a genome sequence. The predictive identification of several hundred CAZyme encoding genes in the *P. sclerotigenum* genome agrees with the observed size and CAZymes numbers within other *Penicillium* spp. (and *Aspergillus* spp.) (Barrett et al., 2020b), and the pectinolytic enzyme data (Tables 5, 6) concur with gene expression results reported for *Aspergillus niger* (Kowalczyk et al., 2017; Alazi et al., 2018), but as hypothesized, the *P. sclerotigenum* also harbors genes that appear to encode additional CAZymes of interest in relation to complex pectin degradation. Among those, there was one GH0 enzyme which might be associated with pectin degradation. By explorative proteomics analysis, we discovered an additional five abundant proteins in the exo-proteome that did not match any known CAZymes proteins or other known protein domains. The data suggest that *P. sclerotigenum* may harbor genes encoding novel proteins of significance in pectin utilization, especially sweet potato pulp pectin. Any such use would require the genes to be cloned and the proteins to be heterologously expressed in a high protein yielding fungal “workhorse” production host, such as *A. niger* or *A. oryzae*, which have a long history of safe use in industrial enzyme production. The results reported may therefore have significant implications for targeted selective modification of pectin. In general, the versatile strategy combining explorative proteomics with CUPP bioinformatics secretome analysis of fungal CAZymes can help elucidate features of fungal carbohydrate substrate degradation and is notably an efficient route for targeted enzyme discovery.

## Data Availability

The datasets presented in this study can be found in online repositories. The names of the repository/repositories and accession number(s) can be found at European Nucleotide Archive PRJEB50413.
